# Reactive oxygen species-dependent mitochondrial dynamics and autophagy confer protective effects in retinal pigment epithelial cells against sodium iodate-induced cell death

**DOI:** 10.1186/s12929-019-0531-z

**Published:** 2019-05-22

**Authors:** Chi-Ming Chan, Duen-Yi Huang, Ponarulselvam Sekar, Shu-Hao Hsu, Wan-Wan Lin

**Affiliations:** 1 Department of Pharmacology, College of Medicine, National Taiwan University, Taipei, Taiwan; 2 Department of Ophthalmology, Cardinal Tien Hospital, New Taipei City, Taiwan; 3 School of Medicine, Fu Jen Catholic University, New Taipei City, Taiwan; 4 Graduate Institute of Medical Sciences, Taipei Medical University, Taipei, Taiwan

**Keywords:** Retinal epithelium, Reactive oxygen species, Sodium iodate, Mitochondrial dynamics, Autophagy, Age-related macular degeneration

## Abstract

**Background:**

Oxidative stress is a major factor in retinal pigment epithelium (RPE) cells injury that contributes to age-related macular degeneration (AMD). NaIO3 is an oxidative toxic agent and its selective RPE cell damage makes it as a reproducible model of AMD. Although NaIO3 is an oxidative stress inducer, the roles of ROS in NaIO3-elicited signaling pathways and cell viability have not been elucidated, and the effect of NaIO3 on autophagy in RPE cells remains elusive.

**Methods:**

In human ARPE-19 cells, we used Annexin V/PI staining to determine cell viability, immunoblotting to determine protein expression and signaling cascades, confocal microscopy to determine mitochondrial dynamics and mitophagy, and Seahorse analysis to determine mitochondrial oxidative phosphorylation.

**Results:**

We found that NaIO3 can dramatically induce cytosolic but not mitochondrial ROS production. NaIO3 can also activate ERK, p38, JNK and Akt, increase LC3II expression, induce Drp-1 phosphorylation and mitochondrial fission, but inhibit mitochondrial respiration. Confocal microscopic data indicated a synergism of NaIO3 and bafilomycin A1 on LC3 punctate formation, indicating the induction of autophagy. Using cytosolic ROS antioxidant NAC, we found that p38 and JNK are downstream signals of ROS and involve in NaIO3-induced cytotoxicity but not in mitochondrial dynamics, while ROS is also involved in LC3II expression. Unexpectedly NAC treatment upon NaIO3 stimulation leads to an enhancement of mitochondrial fragmentation and cell death. Moreover, inhibition of autophagy and Akt further enhances cell susceptibility to NaIO3.

**Conclusions:**

We conclude that NaIO3-induced oxidative stress and cytosolic ROS production exert multiple signaling pathways that coordinate to control cell death in RPE cells. ROS-dependent p38 and JNK activation lead to cytotoxicity, while ROS-mediated autophagy and mitochondrial dynamic balance counteract the cell death mechanisms induced by NaIO3 in RPE cells.

## Introduction

Age-related macular degeneration (AMD) is characterized by progressive degenerative change of the macula of the retina, resulting in loss of central vision. The retinal pigment epithelium (RPE), a monolayer of pigmented cells, is located between photoreceptor cells and Bruch’s membrane and plays essential functional roles for maintenance of retinal homeostasis and for support and health of photoreceptors [1]. Although the pathophysiology of AMD is not well understood, one of the risk factors associated with it is oxidative stress injury of the RPE [2, 3]. Thereby, cumulative and prolonged oxidative damage of the RPE may contribute to development of AMD.

Autophagy is a conserved cell survival pathway that catabolizes damaged proteins and organelles to maintain homeostasis. Through recruitment of selective cargo, formation of specific autophagic vesicles (as named as autophagosome) and lysosomal degradation, autophagy serves as a potential source of nutrients. However, depending on the plasticity of autophagy in response to different stimuli and cellular contexts, autophagy can modulate cell viability, either enhancing survival or death [4]. The aged retina is characterized by increased levels of reactive oxygen species (ROS), impaired autophagy, excessive energy consumption and DNA damage, all of which contribute to the degeneration of RPE cells and link to AMD pathogenesis [5–7].

Sodium iodate (NaIO3) is an oxidative toxic agent and its selective RPE cell damage makes it as reproducible in vitro and in vivo models of AMD [8–10]. At lethal concentration (≥10 mM), NaIO3 was reported to induce RPE cell death involving executional caspase 3/7/8-dependent apoptosis and caspase-independent cell necroptosis [11–13]. A synergistic protective effect against NaIO3-induced cell death was observed when co-treating RPE cells with necrostatin-1 (Nec-1) (a RIP1 inhibitor) and Z-VAD (a pan-caspase inhibitor). It was demonstrated that cell death induced by NaIO3 was preceded by mitochondrial dysfunction [14]. To date, the effect of NaIO3 on autophagy in RPE cells and the role of autophagy in NaIO3-induced RPE cell death are still not well clarified. NaIO3 has been shown to increase expression of LC3B-II in RPE cells at 5 mM [15], but block autophagic flux and mitochondrial-lysosomal axis at lower concentration (1 mM), leading to increased lipofuscinogenesis and impaired phagocytic activities. These results seem to be controversial, and need further investigation. Moreover, although NaIO3 is an oxidative stress inducer, the roles of ROS in NaIO3-elicited signaling pathways and cell viability control have not been clearly elucidated.

In this study using human RPE cell line ARPE-19, we found that NaIO3 can induce cytosolic ROS production, which regulates mitochondrial dynamics, mediates autophagy and activates stress kinases (p38 MAPK and JNK). Scavenging ROS by NAC and Trolox unexpectedly enhances cell death in response to NaIO3. We conclude that p38 MAPK and JNK activation contribute to NaIO3-induced cytotoxicity, while autophagy, Akt activation and cytosolic ROS-dependent mitochondrial dynamics are involved to counteract the cell death mechanisms induced by NaIO3 in RPE cells.

## Materials and methods

### Reagents

NaIO3, N-acetyl cysteine (NAC), Nec-1, dichlorodihydrofluorescein diacetate (H_2_DCFDA), MitoSOX, propidium iodide (PI), oligomycin, carbonyl cyanide-p-trifluoromethoxyphenylhydrazone (FCCP), rotenone, antimycin A, bafilomycin A1 (Baf A1), U0126*,* SP600125, SB203580, Akt inhibitor (AktI), 3-methyladenine (3-MA), and Trolox were obtained from Sigma-Aldrich Co (St Louis, MO, USA). The antibodies specific for phospho-ERK1/2 (T202/Y204), ERK1/2, phospho-JNK (T183/Y185), JNK, phospho-p38 (T180/Y182), p38, phospho-dynamin-related protein (DRP)-1 (S616), DRP-1, poly(ADP-ribose) polymerase 1 (PARP1), γ-H2AX, LC3, p62, and Tom 20 were purchased from Cell Signaling Technology (Beverly, MA, USA). The antibody specific for β-actin was purchased from Santa Cruz Biotechnology (Santa Cruz, CA, USA). The antibodies specific for mitofusin (MFN)-1, MFN-2, optic atrophy 1 (OPA-1), phospho-Akt (T308) and Akt were purchased from Abcam (Cambridge, UK). Dulbecco’s Modified Eagle’s Medium/Nutrient Mixture F-12 (DMEM/F12), trypsin-EDTA, penicillin, ampicillin and streptomycin were from Invitrogen (Rockville, MD, USA). The ECL reagent (Western blotting lightening chemiluminescence reagent plus) was purchased from PerkinElmer (Wellesley, MA, USA).

### Cell culture

Adult human RPE cell line ARPE-19 was purchased from Food Industry Research and Development Institute (Hsinchu, Taiwan). These cells were maintained in DMEM/F12 supplemented with 10% fetal calf serum (GibcoBRL, Invitrogen Life Technologies, Carlsbad, CA, USA), 100 units/ml penicillin and 100 μg/ml streptomycin (Sigma-Aldrich Co.). The cells were cultured in a humidified incubator at 37 °C and 5% CO_2_. For most of the experiments, cells reaching a 90–95% of confluence were starved and synchronized in serum-free DMEM for 24 h before they were subjected to further analysis.

### Annexin V-FITC/PI assay

The cell surface exposure of phosphatidylserine and the plasma membrane impairment of cells were assessed using Annexin V-FITC Apoptosis Detection Kit (Calbiochem). Briefly, suspension of treated/control ARPE-19 cells, containing 5 × 10^5^ cells, was washed with PBS and re-suspended in 0.5 ml cold binding buffer. Then, 1.25 μl of Annexin V-FITC was added and the cells were incubated in the dark for 15 min at room temperature. Following incubation, the cells were centrifuged at 100×*g* for 5 min and the supernatant was removed. The cell pellet was re-suspended in 0.5 ml cold binding buffer, and 10 μl of the 30 μg/ml propidium iodide (PI) solution was added. Cell samples were placed on ice, away from light, and FITC and PI fluorescence were immediately measured by using flow cytometer (Cytomics FC500; Beckman-Coulter, Brea, CA, USA). Data were analyzed using Cell Quest Pro software (Becton Dickinson, Franklin Lakes, NJ, USA). The populations of live cells, early apoptotic cells, late apoptotic and necrotic cells were determined.

### Determination of the cytosolic ROS and mitochondrial ROS

Cytosolic ROS production was detected using H_2_DCFDA for H_2_O_2_ and mitochondrial ROS was detected using mitoSOX. After drug treatment, ARPE-19 cells were washed with PBS and incubated with 10 μM H_2_DCFDA or 5 μM MitoSOX Red at 37 °C for 30 min. Subsequently, the cells were washed in PBS, trypsinized and the fluorescence intensity was measured by flow cytometry (Cytomics FC500; Beckman-Coulter) at excitation/emission wavelengths of 485/530 nm and 510/580 nm for DCFDA and mitoSOX, respectively. For each sample, ROS production was expressed as mean fluorescence ratio (fluorescence of exposed cells/fluorescence of control cells) from the same experiment.

### Cell lysate preparation and Western blot analysis

After stimulation, the medium was aspirated. Cells were rinsed twice with ice-cold PBS, and 25–100 μl of cell lysis buffer (20 mM Tris–HCl, pH 7.5, 125 mM NaCl, 1% Triton X-100, 1 mM MgCl_2_, 25 mM β-glycerophosphate, 50 mM NaF, 100 μM Na_3_VO_4_, 1 mM PMSF, 10 μg/ml leupeptin and 10 μg/ml aprotinin) was then added to each well. After harvesting, cell lysates were sonicated and centrifuged, and equal protein amounts of soluble protein, as determined by the Bradford protein assay, were denatured, subjected to sodium dodecylsulfate polyacrylamide gel electrophoresis (SDS-PAGE), and transferred to a polyvinylidene difluoride membrane. Non-specific binding was blocked with TBST (50 mM Tris-HCl, pH 7.5, 150 mM NaCl and 0.02% Tween 20) containing 5% non-fat milk for 1 h at room temperature. After immunoblotting with the first specific antibody, membranes were washed three times with TBST and incubated with a horseradish peroxidase (HRP) conjugated secondary antibody for 1 h. The dilution folds of first specific antibodies and β-actin were 1:1000 and 1:10,000, respectively. After three washes with TBST, the protein bands were detected with enhanced chemiluminescence detection reagent. To make sure equal amounts of sample protein applied for electrophoresis and immunoblotting, β-actin was used as an internal control.

### Measurement of mitochondrial oxygen consumption rate

The oxygen consumption rate (OCR) was measured by the extracellular flux analyzer XF24 (Seahorse Bioscience, Houston, TX, USA) as we previously described [16]. Cells were plated at 4 × 10^5^ cells/well in a Seahorse 24-well V7 microplate (Seahorse Bioscience) and cultured in complete DMEM growth medium for 24 h in a 5% CO_2_ incubator at 37 °C. Then, the medium was removed and cells were incubated in XF assay medium in the absence of NaHCO_3_ and FBS for 1 h at 37 °C in measuring chamber without CO_2_ input. The mitochondrial complex inhibitors (oligomycin, FCCP, antimycin A1/ rotenone) were freshly prepared in XF assay media. After 26 min of measuring the basal respiration, oligomycin (2.5 μM) was injected, followed by FCCP (1 μM) at 50 min, and antimycin A (2.5 μM) /rotenone (2.5 μM) at 74 min. OCR was recorded as pMoles per minute. ATP turnover was measured after the treatment with oligomycin. Averages of three wells were taken per data point.

### Mitochondrial imaging

Cells were initially fixed with 4% paraformaldehyde at 37 °C followed by permeabilization with 0.2% Triton X-100 for 15 min, and blocking by BSA (5%) and normal IgG (1:300) for 1 h. For mitophagy measurement, immunostaining was then performed using primary antibody against Tom20 or LC3 (Abcam, Cambridge, UK) in 1% BSA overnight at 4 °C. After washing with PBS, cells were incubated with secondary antibody in 1% BSA in PBS for 1 h at room temperature and then mounted with DAPI Fluoromount-G (SouthernBiotech, Birmingham, AL, USA). Images were acquired using a 100 X Plan-Neofluar oil objective of LSM 880 with Airyscan SR microscopy (Carl Zeiss Micro Imaging GmbH, Jena, Germany). The co-localization of Tom20 (marker of mitochondria) and LC3 (marker of autophagosome) was determined by Zen co-localization software and on a pixel by pixel basis. Every pixel in the image was plotted in the scatter diagram based on its intensity level from each channel. The co-localization coefficients were measured for each channel.

### Statistical analysis

All data were obtained from at least three separate experiments and presented as mean ± standard error mean (S.E.M.). Analysis of variance was used to assess the statistical significance of the differences. A *p* value of less than 0.05 was considered statistically significant.

## Results

### NaIO3-induced mixed type cell death in ARPE-19 cells is accompanied by ROS production and mitochondrial dysfunction

**Fig. 1:**
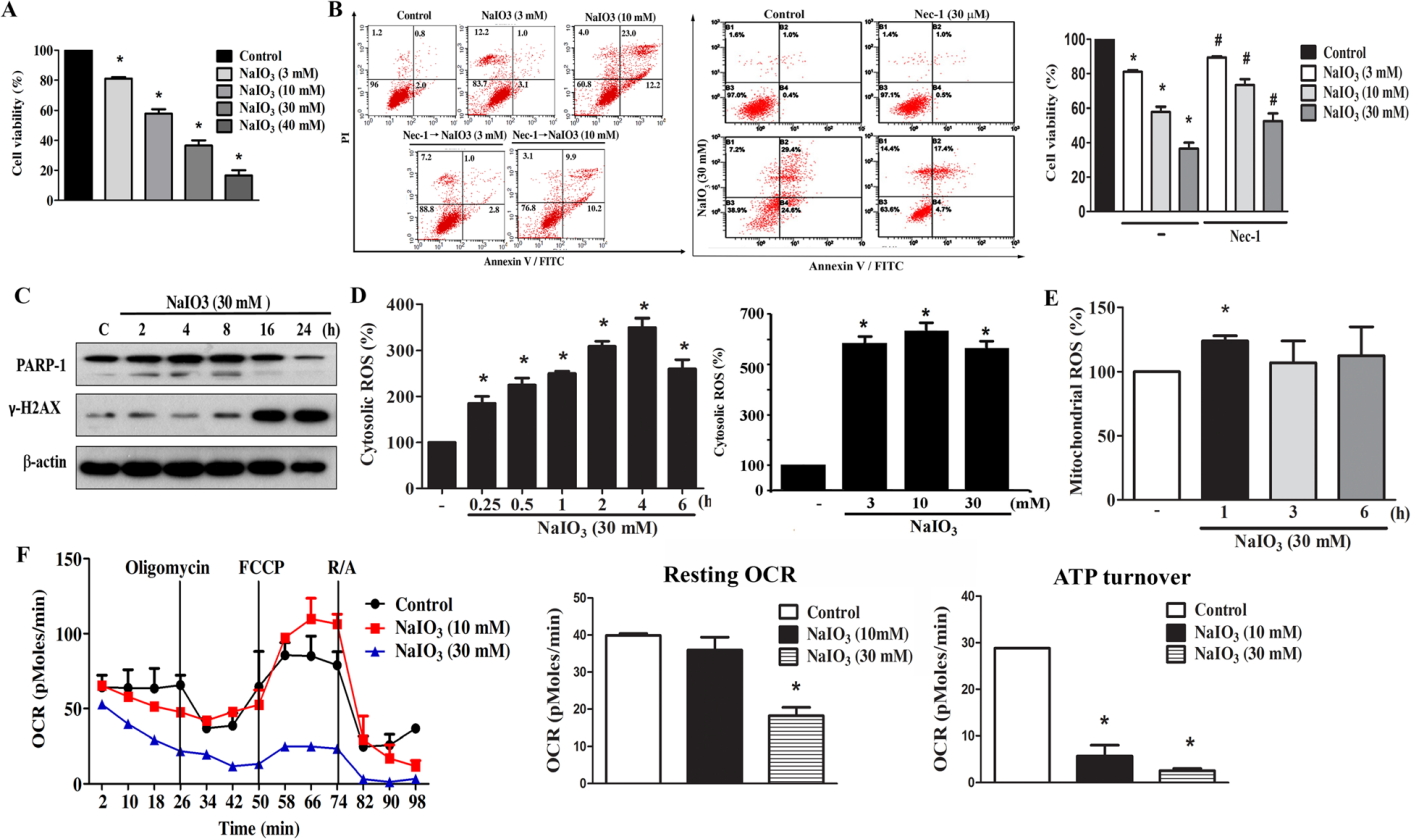
NaIO_3_ induces cell death, ROS production and mitochondrial respiratory inhibition. **a** Cells were treated with NaIO3 (3, 10, 30, 40 mM) for 24 h. **b** Cells were pretreated with Nec-1 (30 μM) 15 min prior to NaIO3 (3, 10, 30 mM) treatment for 24 h. Cell viability was determined by Annexin V/PI staining and flow cytometry. **c** After treatment with NaIO3 (30 mM) for different periods, cell lysates were collected for immunoblotting of PARP1 and γ-H2AX. **d**, **e** Cells were treated with NaIO3 (30 mM) for different periods (**d**, left panel and **e**) or at different concentrations for 3 h (**d**, right panel). Cytosolic ROS **d** and mitochondrial ROS **e** were determined by H_2_DCFDA and MitoSOX, respectively. **f** Cells were treated with 10 and 30 mM NaIO3 for 24 h, followed by measuring OCR status by Seahorse Bioscience XF24 Analyzer as indicated in the Methods. Data were mean ± S.E.M. from three independent experiments. * *p* < 0.05, indicating significant effect of NaIO3 on inducing cell death (**a**, **b**), ROS production (**d**, **e**) and inhibition of mitochondrial respiration (**f**) as compared to vehicle-treated control group. # *p* < 0.05, indicating the significant effect of Nec-1 to reduce NaIO3-induced cytotoxicity shown in (**b**)

Before addressing the signaling cascades that mediate the cell death caused by NaIO3, we first clarified the death mode. We found that NaIO3 can induce a concentration-dependent cell death in RPE cells with IC_50_ of 20 mM (Fig. 1a). Nec-1, the selective inhibitor of RIP1, only partially protects cells against NaIO3 (10 or 30 mM)-induced cytotoxicity, implying both necroptosis and other death mode are involved under NaIO3 treatment (Fig. 1b). In addition, we found that NaIO3 at 30 mM can induce PARP1 cleavage (an index of activation of executional caspase 3) and γ-H2AX expression (an index of DNA damage) (Fig. 1c). These findings suggest a mixed type cell death is induced by NaIO3.

In agreement with previous findings that NaIO3 is an oxidative stress inducer in RPE [8, 17, 18], our data revealed increases of cytosolic and mitochondrial ROS after NaIO3 (30 mM) treatment. Of note, the cytosolic ROS was rapidly increased at 15 min and reached the plateau of about 3-fold increase within 2–6 h (Fig. 1d, left panel). NaIO3 at low concentration (3 mM) was sufficient to induce similar extent of cytosolic ROS increase as 30 mM after 3 h treatment (Fig. 1d, right panel). In contrast, mitochondrial ROS level was only transiently increased by NaIO3 (30 mM) at 1 h and the increased extent was small of around 25% (Fig. 1e). Using Seahorse equipment and flux analysis, we measured the OCR. Our data revealed that NaIO3 treatment at 10 and 30 mM for 24 h led to a dramatic inhibition of basal OCR and ATP turnover (Fig. 1f).

### Antioxidant NAC and Trolox enhance NaIO3-induced mitochondrial fission and cell death

**Fig. 2:**
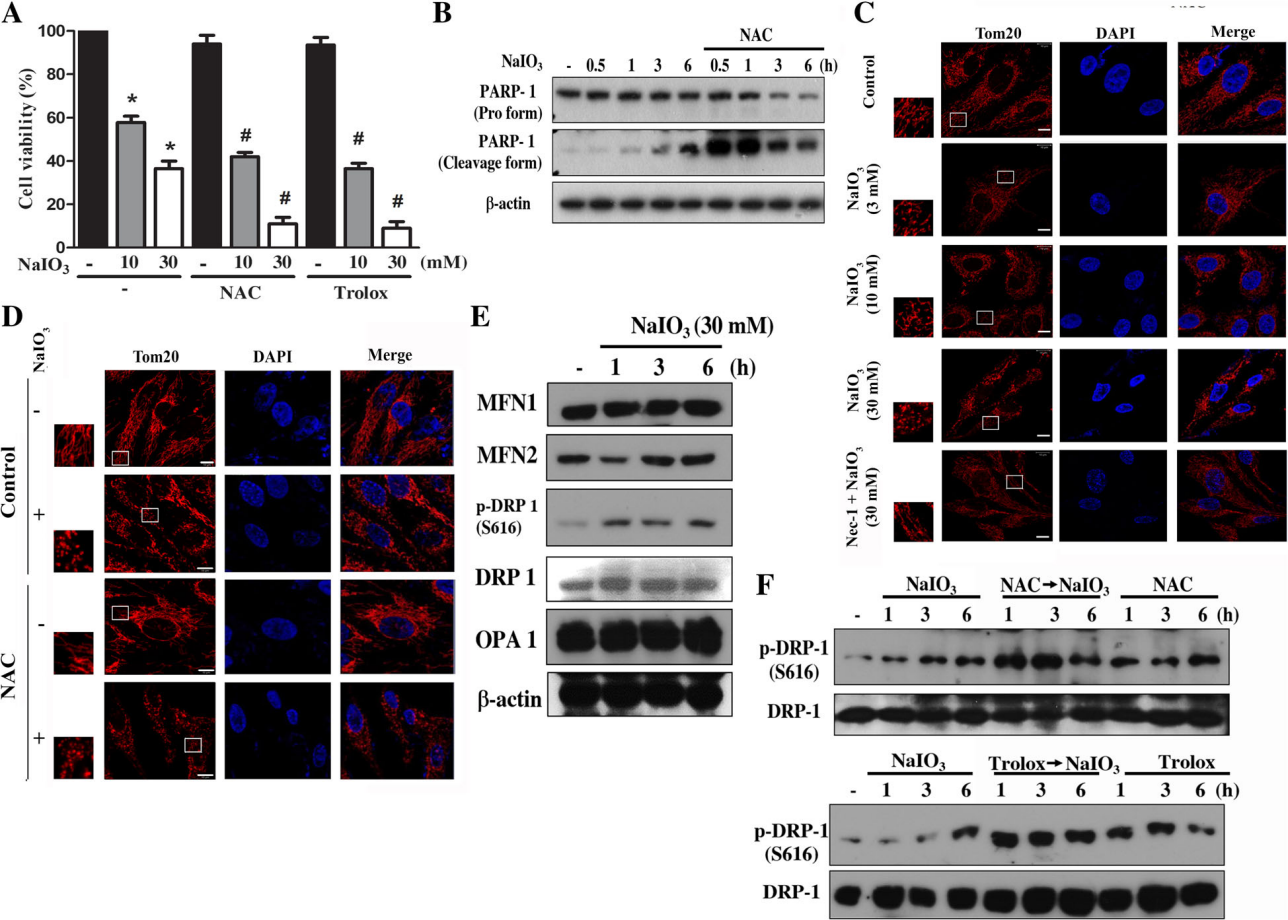
Antioxidant enhanced mitochondrial fission and cell death in NaIO3-treated RPE cells. **a** Cells were treated with NAC (10 mM), Trolox (10 mM) and NaIO3 (10, 30 mM) for 24 h. Cell viability was determined by Annexin V/PI staining and flow cytometry. Data were mean ± S.E.M. from three independent experiments. * *p* < 0.05, indicating significant cytotoxic effect of NaIO3. # *p* < 0.05, indicating significant effects of NAC and Trolox. **b** After NAC and/or NaIO3 treatment, PARP1 protein was determined by immunoblotting. **c**, **d** Cells were treated with Nec-1 (30 μM) (**c**) or NAC (10 mM) (**d**) followed by NaIO3 at 3–30 mM (**c**) or 30 mM (**d**) for 6 h. Afterwards cells were stained with Tom20 (indicator of mitochondria) to determine mitochondrial shape. Scale bars indicated 10 μm. **e**, **f** Cells were treated with NAC (10 mM), Trolox (10 mM) and/or NaIO3 (30 mM) as indicated, and immunoblotting was used to determine MFN1/2, Drp-1, and OPA1

Because oxidative stress has been identified as a major inducer of RPE injury, which eventually leads to a loss of vision, we interested to understand the role of cytosolic ROS that was increased under NaIO3 stimulation in cell viability. Therefore, we determined the effect of antioxidant NAC and Trolox. As shown in Fig. 2a, NAC (10 mM) and Trolox (10 mM) cannot inhibit NaIO3 (10, 30 mM)-induced cytotoxicity, and unexpectedly both treatments further enhanced the death effect of NaIO3. Immunoblotting data further confirmed this finding, as NaIO3-induced PARP1 cleavage (an index of caspase 3 activation) was increased by NAC (Fig. 2b). All these results suggest that although oxidative stress is induced by NaIO3, ROS might exert protective signaling pathways to counteract cell death.

In addition, although mitochondrial dysfunction contributes to NaIO3-induced cytotoxicity as previously reported [14] and our above findings, to date there are no reports regarding the effect of NaIO3 on mitochondrial morphology. Therefore, we determined the effect of NaIO3, either in the absence or presence of NAC and Nec-1, on mitochondrial dynamics. Using confocal microscopy, we found that mitochondria became severe fragmented and fission after 6 h treatment with 30 mM NaIO3. Lower concentrations of NaIO3 (3, 10 mM) did not significantly change mitochondrial dynamics at 6 h (Fig. 2c). Of note this effect of 30 mM NaIO3 was prevented by Nec-1 (Fig. 2c), but enhanced by NAC. NAC itself did not alter the mitochondrial morphology (Fig. 2d). To confirm the effect of NaIO3 on mitochondrial fission, we determined several key molecules that help to regulate the shape of mitochondria. We found that NaIO3 did not change the protein levels of mitofusion 1 and 2 (MFN1/2) nor OPA1, which are major regulators for mitochondrial fusion. In contrast, although Drp-1 protein level was not changed, its Ser616 phosphorylation level, which is an index of mitochondrial fission, was rapidly increased by NaIO3 (Fig. 2e). In agreement with the exacerbated effect of NAC on mitochondria fission, Drp-1 (S616) phosphorylation was markedly increased under NAC plus NaIO3 co-treatment as compared to NaIO3 treatment alone. Similarly, Trolox also induced the same effect as NAC (Fig. 2f). All these data suggest that ROS-dependent effect on balancing mitochondrial dynamic is present in RPE cells. Mitochondria are prone to fission state when intracellular ROS level is reduced.

### ROS-dependent autophagy protects RPE cells against NaIO3-induced cell death

**Fig. 3:**
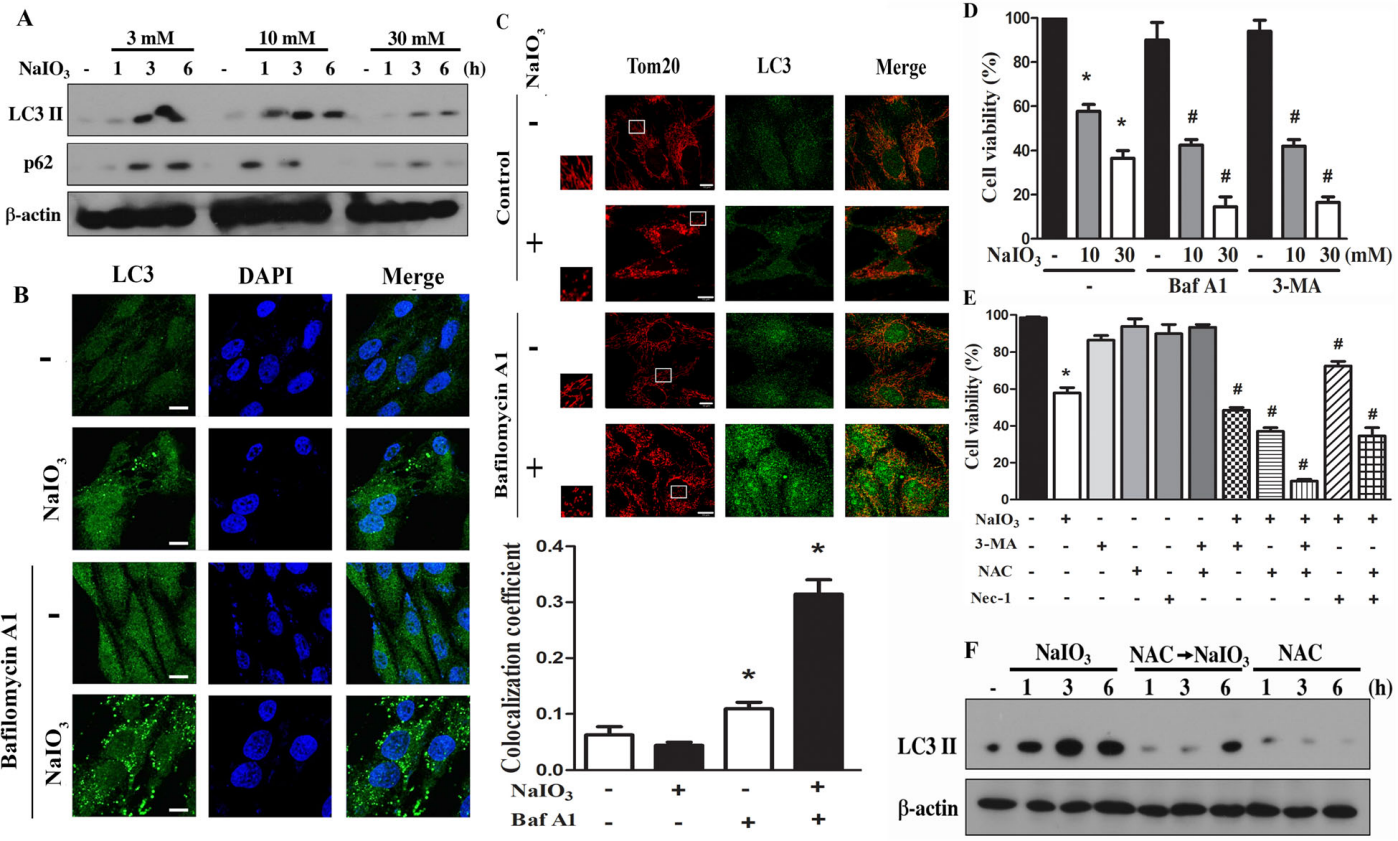
ROS mediate autophagy and exert a survival action in NaIO3-treated RPE cells. **a** After NaIO3 treatment as indicated cell lysates were used to determine LC3 and p62 by immunoblotting. **b**, **c** After treatment with Baf A1 (100 nM) and/or NaIO3 (30 mM) for 6 h, confocal microscopy was used to determine LC3II and Tom20. Scale bars indicated 10 μm. * *p* < 0.05, indicating significant effect of Baf A1, either in the absence or presence of NaIO3. **d**, **e** After treatment with drugs as indicated (i.e. 100 nM Baf A1, 3 mM 3-MA, 10 mM NAC, 30 μM Nec-1, 10 or 30 mM NaIO3) for 24 h, cell viability was determined. Data were mean ± S.E.M. from three independent experiments. * *p* < 0.05, indicating significant cytotoxic effect of NaIO3. # *p* < 0.05, indicating significant effects of Baf A1, 3-MA, and NAC on the action of NaIO3. **f** After treatment with NAC (10 mM) and/or NaIO3 (30 mM), LC3 protein was determined by immunoblotting

Because ROS-dependent autophagy is a key process to maintain cell homeostasis, survival and phagocytic function of RPE cells [19, 20], we examined the concentration- and time-dependent effects of NaIO3 on autophagy. From immunoblotting, we observed that NaIO3 can time-dependently increase LC3II accumulation at 3 mM and 10 mM, while this effect became weak at 30 mM (Fig. 3a). We speculate the less LC3II effect at 30 mM than that at 3–10 mM might be due to other events associated with cytotoxicity. Concomitantly NaIO3 increased p62 expression of higher extent at 3 and 10 mM than 30 mM (Fig. 3a). Strengthening the ability of NaIO3 to induce autophagic flux, the confocal microscopic data revealed that LC3 staining was increased by NaIO3 and bafilomycin A1 individually after 6 h treatment, and co-treatment of both dramatically enhanced this effect (Fig. 3b). The synergistic effects of NaIO3 and bafilomycin A1 on LC3 punctate indicate the autophagic flux induced by NaIO3. Moreover, when co-staining cells with Tom20 and LC3 to indicate the feature of mitophagy, our data revealed that NaIO3 and bafilomycin A1 co-treatment can increase the signal co-localization (Fig. 3c). These findings suggest the efficient induction and rapid turnover rate of mitophagy in NaIO3-treated RPE cells. These findings together with above notions allow us to suggest that severe mitochondrial fission and mitochondrial dysfunction contribute to NaIO3-induced cell death.

To further understand the role of autophagy in RPE cell viability, we used 3-MA, an inhibitor of class III PI3K and subsequent autophagic flux initiation. We found that the cell death extent caused by NaIO3 was enhanced by 3-MA (Fig. 3d), indicating autophagy constitutes a protection mechanism in RPE cells. Likewise, bafilomycin A1 also increased cell susceptibility to NaIO3 (Fig. 3d). When co-treating NAC and 3-MA, an additive effect on cell death was observed (Fig. 3e). In addition, the effect of NAC on increasing cell death was also observed in cells treated with Nec-1 (Fig. 3e). To further understand the link between NAC enhancement of cell death and cell protective function of autophagy, we determined the effect of NAC on autophagy in RPE cells before and after NaIO3 stimulation. As shown in Fig. 3f, NAC can reduce NaIO3 (30 mM)-induced LC3II accumulation. These findings suggest that ROS might play an important role to mediate protective autophagy in RPE cells, and ROS production under NaIO3 stimulation also reduces mitochondrial fission.

### ROS mediates NaIO3-induced p38 and JNK activation, but not ERK activation

**Fig. 4:**
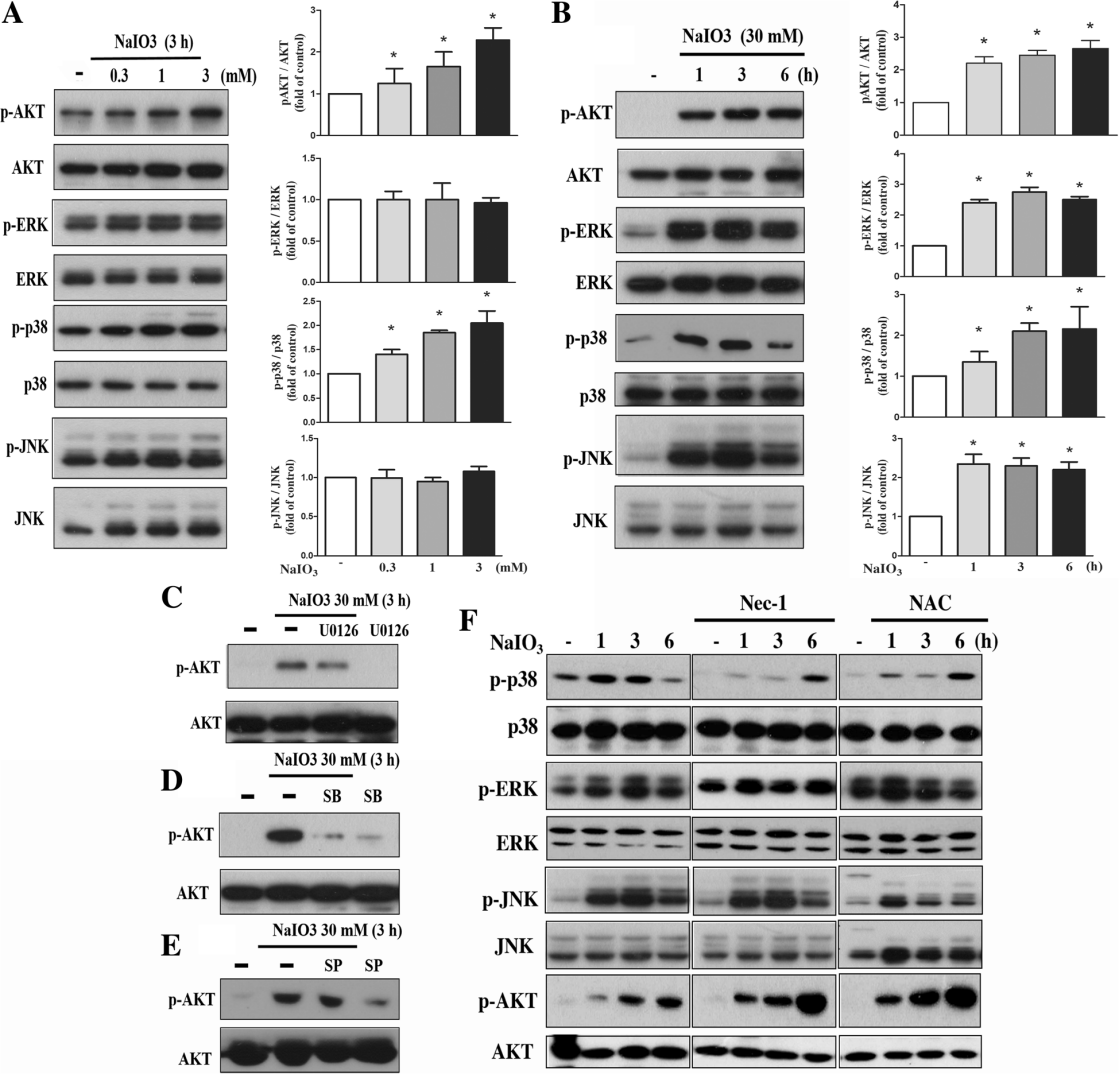
NaIO3 induces Akt, ERK, JNK and p38 MAPK activation. **a**, **b** As indicated, cells were treated with NaIO3 at concentrations indicated for different intervals. Immunoblotting was conducted by specific antibodies to determine the total and phosphorylated forms of Akt, ERK, p38 MAPK  and JNK. Quantification of protein phosphorylation was determined by normalization with respective total protein levels. * *p* < 0.05, indicating significant activation effects of NaIO3 on signaling pathways. **c**-**f** Cells were pretreated with U0126 (ERK inhibitor, 10 μM) (**c**), SB203580 (p38 MAPK inhibitor, 10 μM) (**d**), SP600125 (JNK inhibitor, 10 μM) (**e**), Nec-1 (RIP1 inhibitor, 30 μM) (**f**) or NAC (10 mM) (**f**) for 15 min. Then cells were treated with NaIO3 (30 mM) for 3 h (**c**-**e**) or different time intervals (**f**). Cell lysates were collected for immunoblotting

Next we like to understand the signaling pathways contributing to ROS-dependent effects on cell viability under NaIO3 stimulation. We found that NaIO3 (3 mM) can increase Akt phosphorylation after 3 h treatment. p38 MAPK phosphorylation was slightly increased. In contrast, JNK and ERK activities were not altered by NaIO3 at 3 mM (Fig. 4a). Increasing concentration up to 30 mM, we found the abilities of NaIO3 to rapidly and dramatically increase Akt, ERK, and JNK activation (Fig. 4b). After observing the activation of Akt, ERK, JNK and p38 MAPK by 30 mM NaIO3, we determined the potential crosstalk of signaling cascades by pharmacological inhibitors. We found that U0126 (ERK inhibitor) and SB203580 (p38 MAPK inhibitor) can significantly inhibit NaIO3-elicited Akt activation (Fig. 4c, d), while SP600125 (JNK inhibitor) had no effect (Fig. 4e).

Because RIP1-dependent necroptosis was involved in the cell death action of NaIO3, we further investigated the role of RIP1 in signaling pathway. We found that Nec-1 can inhibit NaIO3-induced p38 MAPK phosphorylation, but had no effects on ERK and JNK (Fig. 4f). In contrast, an increased NaIO3-induced Akt phosphorylation by Nec-1 was observed (Fig. 4f). In addition, we investigated the link between ROS and signaling pathways. As a result, NAC can inhibit p38 and JNK activation, while cannot affect ERK activation. Unexpectedly NAC can increase Akt activation caused by NaIO3 (Fig. 4f). These findings suggest that NaIO3-induced Akt activation is partially dependent on ROS-RIP1-p38 MAPK pathway. JNK and p38 MAPK but not ERK are downstream signal pathways of ROS.

### NaIO3-induced p38 and JNK activation contribute to cell death

**Fig. 5:**
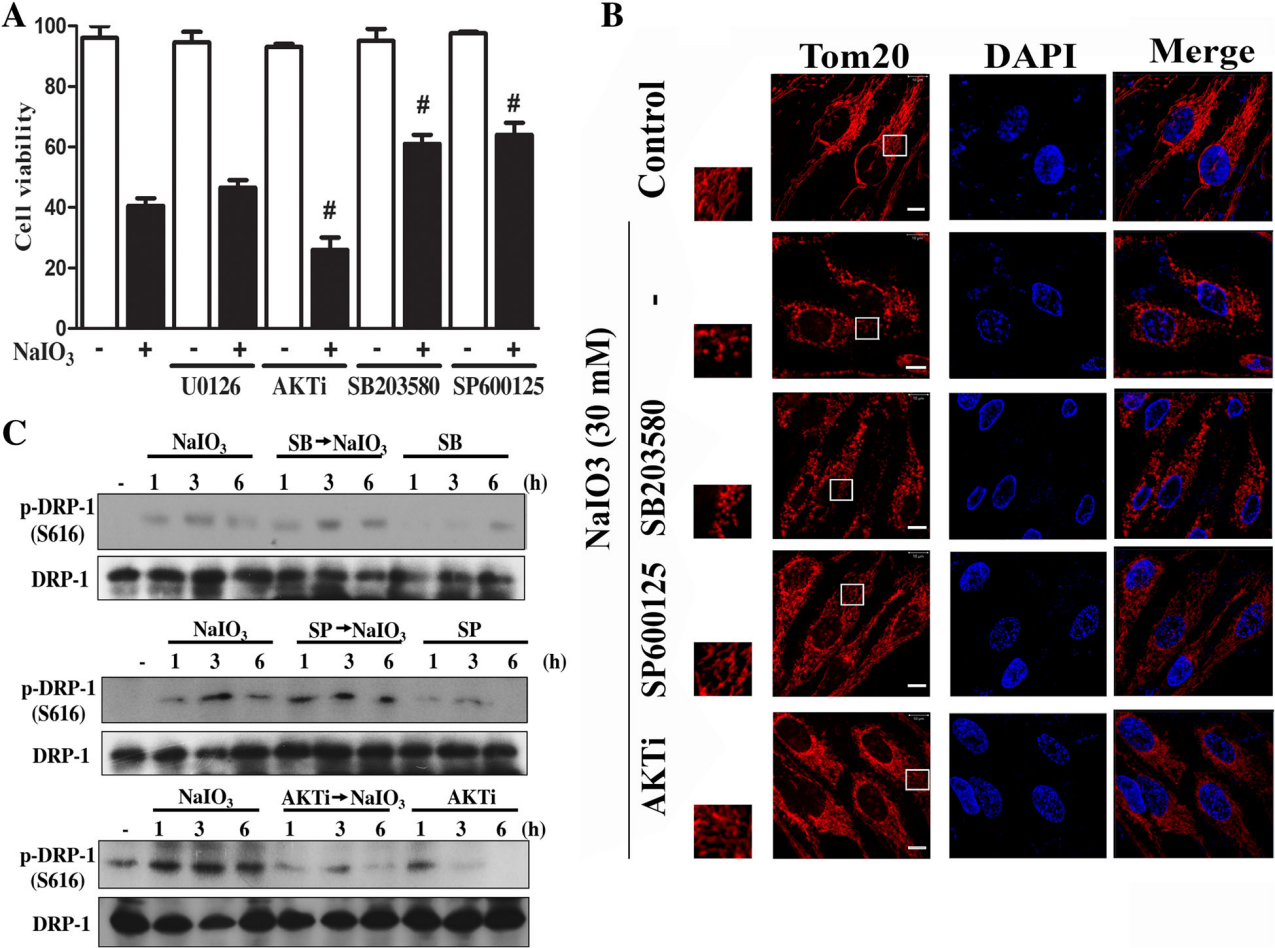
Roles of p38, JNK and Akt in mitochondrial dynamics and cell viability in NaIO3-treated RPE cells. **a** Cells were treated with specific kinase inhibitors (U0126, AktI, SB203580, SP600125) and NaIO3 for 24 h. Cell viability was determined. # *p* < 0.05, indicating significant effects of kinase inhibitors on NaIO3-induced cell death. **b** After drug treatment for 6 h, Tom20 was used to stain mitochondria and confocal microscopy was taken to determine the mitochondrial dynamics. Scale bars indicated 10 μm. **c** After drug treatment as indicated, Drp-1 phosphorylation at S616 was determined by immunoblotting. Data were representative of 3 independent experiments

Next we used the pharmacological inhibitors to understand the roles of signaling protein kinases in NaIO3-induced cytotoxicity. As shown in Fig. 5a, SB203580 and SP600125 can partially protect cells against NaIO3-induced cell death, U0126 had no effect, while Akt inhibitor AktI enhanced the cell death event. In addition, we found that SB203580 and SP600125 did not alter NaIO3-induced mitochondrial fission, while AktI exerts a partial inhibition (Fig. 5b). Accordingly, SB203580 and SP600125 failed to change Drp-1 (S616) phosphorylation induced by NaIO3, while AktI inhibited this response of NaIO3 (Fig. 5c). All these data suggest that activation of stress kinases p38 MAPK and JNK leads to cell death, and this event is not related to mitochondrial dynamics. In contrast, Akt exerts cell protective effect and this event might be related to attenuation of mitochondrial fission under NaIO3 treatment.

## Discussion

NaIO3-induced cell death in RPE cells is a valuable in vitro model of AMD. Although many studies using this model demonstrate the death features and molecular events underlying the oxidative stress-mediated cellular responses mimicking the pathogenesis of AMD, it remains unclear regarding ROS-mediated signaling pathways, mitochondrial function, autophagy and mitophagy that are integrated to control cell viability in RPE. In this study, we found that NaIO3 can dramatically induce cytosolic ROS but not mitochondrial ROS production in ARPE-19 cells. Furthermore, we demonstrated that ROS are involved in various cellular responses, including autophagic flux, mitochondrial dynamic homeostasis, p38 MAPK and JNK activation. The former two events caused by ROS can support cell viability, while activation of p38 MAPK and JNK contribute to cell death. Although Akt activation is downstream of ERK and p38 MAPK and can counteract NaIO3-induced mitochondrial fission, Akt inhibition further enhances the cell death under NaIO3 treatment. Such multifunctional mechanisms mediated by ROS or independent of ROS orchestrate the complicated and dynamic processes that coordinately regulate stress responses and cell survival. This is the reason why we unexpectedly shed a new insight into the deteriorated cell death by ROS scavenger NAC and Trolox treatment in NaIO3-treated RPE cells.

ROS are double-edged molecules, which not only can mediate physiological signaling pathways, but also are major contributors to oxidative stress. In this study, we demonstrate the role of ROS to mediate NaIO3-induced autophagic flux that constitutes a protective mechanism to attenuate cell susceptibility to NaIO3 insult. Previously many studies have highlighted that optimal acceleration of autophagic degradation is important for RPE cell survival [20–22]. Very surprisingly it is not easy to detect high amounts of LC3 punctate in NaIO3-treated RPE cells, when staining with LC3 antibody alone or in combination with mitochondrial marker Tom20. Nevertheless, LC3 punctate was dramatically and synergistically increased by co-treatment with NaIO3 and bafilomycin A1. Therefore, it is logically speculated that the degradation efficiency of autolysosome in RPE cells is sufficiently high, allowing the rapid digestion of cargo containing in autophagosome including mitochondria. Additionally we confirmed previous finding that NaIO3 can increase p62 expression in RPE cells [14]. In this context, even though we did not have data to illustrate its induction mechanism and cellular function in NaIO3-induced cellular responses, p62 may slow down development of the destructive alterations of RPE cells and exert retinoprotective effect in AMD [23]. Moreover, p62 gene upregulation during autophagy induction which enhances autophagic flux was also observed previously [24]. Therefore, it is warrant to further explore the regulation and cellular function of p62 in RPE cells in the future.

In this study we for the first time revealed the roles of MAPKs dependent on ROS or not in NaIO3-elicited cell death pathway. We observed that ROS are involved in NaIO3-induced activation of stress protein kinases p38 MAPK and JNK, but not ERK activation. Our data indicate both pro-apoptotic factors p38 MAPK and JNK are involved in oxidative stress-induced cell death, and the death mechanism is not on the impairment of mitochondrial dynamics. This notion is in agreement with previous findings that H_2_O_2_-induced RPE cells injury to a certain extent is associated with p38 MAPK and JNK signaling pathways [25]. Currently, we found that ERK is not involved in NaIO3-induced cell death, while it mediates Akt signal pathway. This finding of ERK might be explained by the dual functions of oxidative insult-induced ERK activation, which exerts both pro- and anti-apoptosis [26]. As our data of NaIO3, p38 MAPK but not ERK pathway was reported to mediate HIV-1-induced cell death in RPE cells [27].

Moreover, we found that ERK and p38 MAPK, but not JNK are upstream signaling of Akt, which is a common molecule to maintain cell survival. Unlike the effect to mediate p38 MAPK and JNK activation, cytosolic ROS as well as RIP1 activation seem to antagonize NaIO3-induced Akt activation. Our data revealed an increased Akt phosphorylation upon NAC or Nec-1 co-treatment with NaIO3. Here we ruled out the increased Akt activity induced by NAC and Nec-1 is resulting from p38 MAPK and ERK, because both kinase activities are not changed. In addition, we also found the ability of Akt inhibitor to suppress Drp-1 (S616) phosphorylation and mitochondrial fission caused by NaIO3. Overall it is still needed to elucidate how ROS and RIP1 inhibit Akt, and how Akt inhibits Drp-1 (S616) phosphorylation, which in turn reverses the mitochondrial fission change caused by NaIO3. However, it is sure that Akt is involved in autophagy and other survival mechanisms, and these identified cellular actions can support our finding for the enhancement of cell death by Akt inhibitor in RPE cells.

In normal physiological condition, both mitochondrial dynamics which involves the fission and fusion of mitochondrial outer and inner membranes, and mitochondrial oxidative phosphorylation play crucial roles in maintaining cell metabolism and viability. In contrast, mitochondrial dysfunction by impairing mitochondrial biogenesis or dynamics is a key executioner in several age-related neurodegenerative diseases, including AMD [28]. Apart from inducing MAPKs signaling, ROS can timely regulate mitochondrial dynamics and oxidative phosphorylation. It has been shown that changes in the levels of ROS lead to modifications of several proteins implicating in the mitochondrial dynamics [29, 30]. In this study, the findings of NAC to deteriorate NaIO3-induced mitochondrial fragmentation and cytotoxicity suggest the essential role of cytosolic ROS to counteract mitochondrial fragmentation in certain highly stressed conditions. Detailing the molecular mechanisms linking cytosolic ROS signaling to maintain of mitochondrial dynamics remains an unanswered question. However, we speculate that ROS-dependent protective autophagy might be one of mechanisms. This assumption is based on the inhibition of LC3II formation by NAC. For sure, we cannot rule out other mechanisms underlying this effect of NAC.

## Conclusions

Taken together, NaIO3 can specifically increase cytosolic but not mitochondrial ROS in ARPE-19 cells. Several cytosolic ROS targeting events are induced in NaIO3-treated RPE cells. Cytosolic ROS-dependent p38 and JNK activation lead to cell death, while cytosolic ROS-mediated autophagy and balance of mitochondrial dynamics contribute to cell survival. All these findings suggest a special oxidative stress status in NaIO3-treated RPE cells where cytosolic ROS can simultaneously regulate multiple cellular events. Overall, the present study laid a foundation for exploring novel AMD treatment methods in the future.
