# Correction to: Reactive oxygen species-dependent mitochondrial dynamics and autophagy confer protective effects in retinal pigment epithelial cells against sodium iodate-induced cell death

**DOI:** 10.1186/s12929-019-0555-4

**Published:** 2019-09-04

**Authors:** Chi-Ming Chan, Duen-Yi Huang, Ponarulselvam Sekar, Shu-Hao Hsu, Wan-Wan Lin

**Affiliations:** 1 Department of Pharmacology, College of Medicine, National Taiwan University, Taipei, Taiwan; 2 Department of Ophthalmology, Cardinal Tien Hospital, New Taipei City, Taiwan; 3 School of Medicine, Fu Jen Catholic University, New Taipei City, Taiwan; 4 Graduate Institute of Medical Sciences, Taipei Medical University, Taipei, Taiwan

**Correction to: J Biomed Sci**

**https://doi.org/10.1186/s12929-019-0531-z**

After the publication of this article [1], the authors would like to clarify that some immunoblotting data in Figs. 2f, 3a and 4b were obtained from the same samples but individual SDS-PAGE gels. Therefore, the authors would like to add a separate line between these data, i.e. Drp-1 and Drp-1-p in Fig. 2f; LC3I/II and p62 in Fig. 3a and p38-p and p38 in Fig. 4b. The correction figures for the entire Figs. 2, 3 and 4 have been included below.

**Fig. 2:**
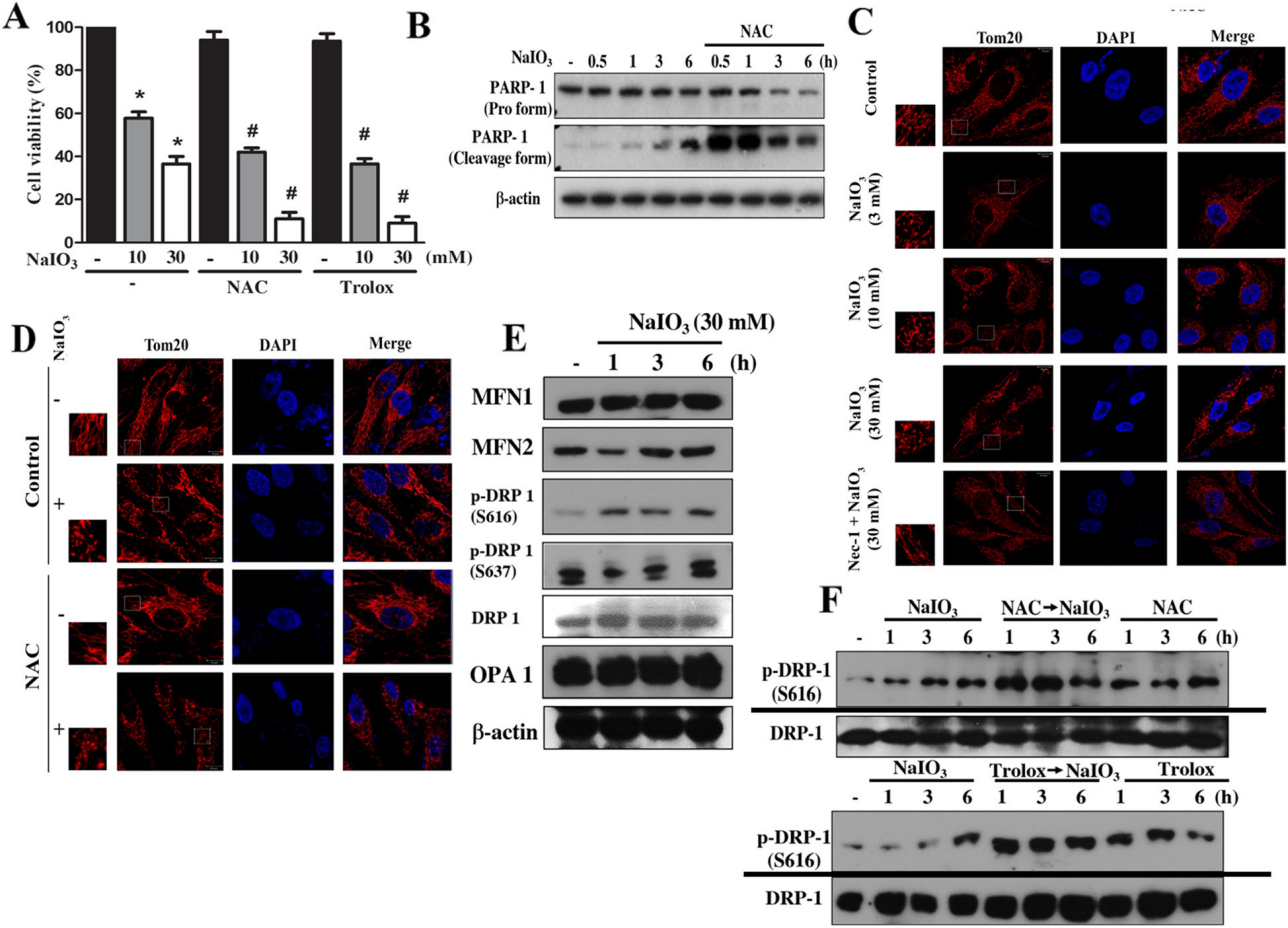
Antioxidant enhanced mitochondrial fission and cell death in NaIO3-treated RPE cells. **a** Cells were treated with NAC (10 mM), Trolox (10 mM) and NaIO3 (10, 30 mM) for 24 h. Cell viability was determined by Annexin V/PI staining and flow cytometry. Data were mean ± S.E.M. from three independent experiments. * *p* < 0.05, indicating significant cytotoxic effect of NaIO3. # *p* < 0.05, indicating significant effects of NAC and Trolox. **b** After NAC and/or NaIO3 treatment, PARP1 protein was determined by immunoblotting. **c**, **d** Cells were treated with Nec-1 (30 μM) (**c**) or NAC (10 mM) (**d**) followed by NaIO3 at 3–30 mM (**c**) or 30 mM (**d**) for 6 h. Afterwards cells were stained with Tom20 (indicator of mitochondria) to determine mitochondrial shape. Scale bars indicated 10 μm. **e**, **f** Cells were treated with NAC (10 mM), Trolox (10 mM) and/or NaIO3 (30 mM) as indicated, and immunoblotting was used to determine MFN1/2, Drp-1, and OPA1

**Fig. 3:**
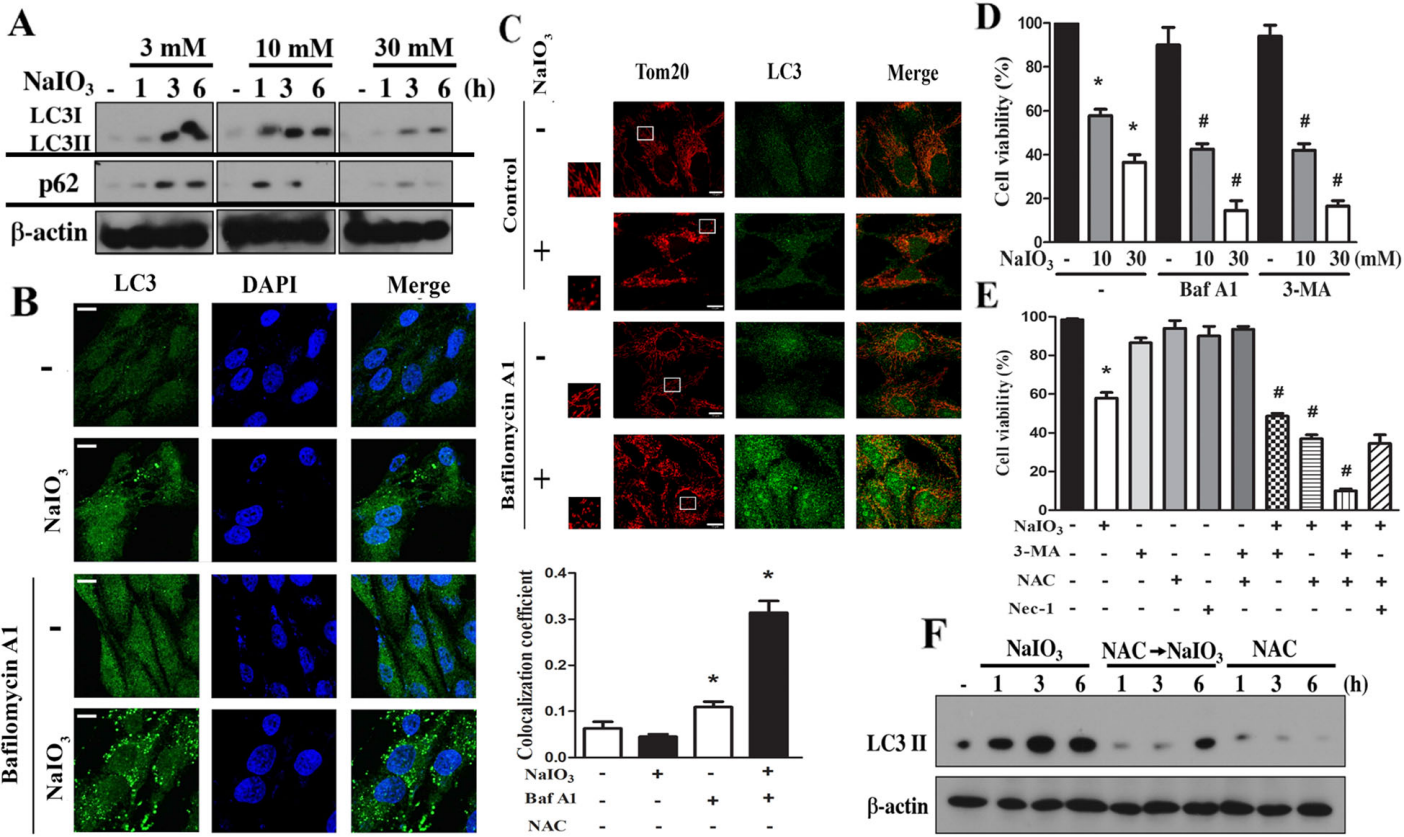
ROS mediate autophagy and exert a survival action in NaIO3-treated RPE cells. **a** After NaIO3 treatment as indicated cell lysates were used to determine LC3 and p62 by immunoblotting. **b**, **c** After treatment with Baf A1 (100 nM) and/or NaIO3 (30 mM) for 6 h, confocal microscopy was used to determine LC3II and Tom20. Scale bars indicated 10 μm. * *p* < 0.05, indicating significant effect of Baf A1, either in the absence or presence of NaIO3. **d**, **e** After treatment with drugs as indicated (i.e. 100 nM Baf A1, 3 mM 3-MA, 10 mM NAC, 30 μM Nec-1, 10 or 30 mM NaIO3) for 24 h, cell viability was determined. Data were mean ± S.E.M. from three independent experiments. * *p* < 0.05, indicating significant cytotoxic effect of NaIO3. # *p* < 0.05, indicating significant effects of Baf A1, 3-MA, and NAC on the action of NaIO3. **f** After treatment with NAC (10 mM) and/or NaIO3 (30 mM), LC3 protein was determined by immunoblotting

**Fig. 4:**
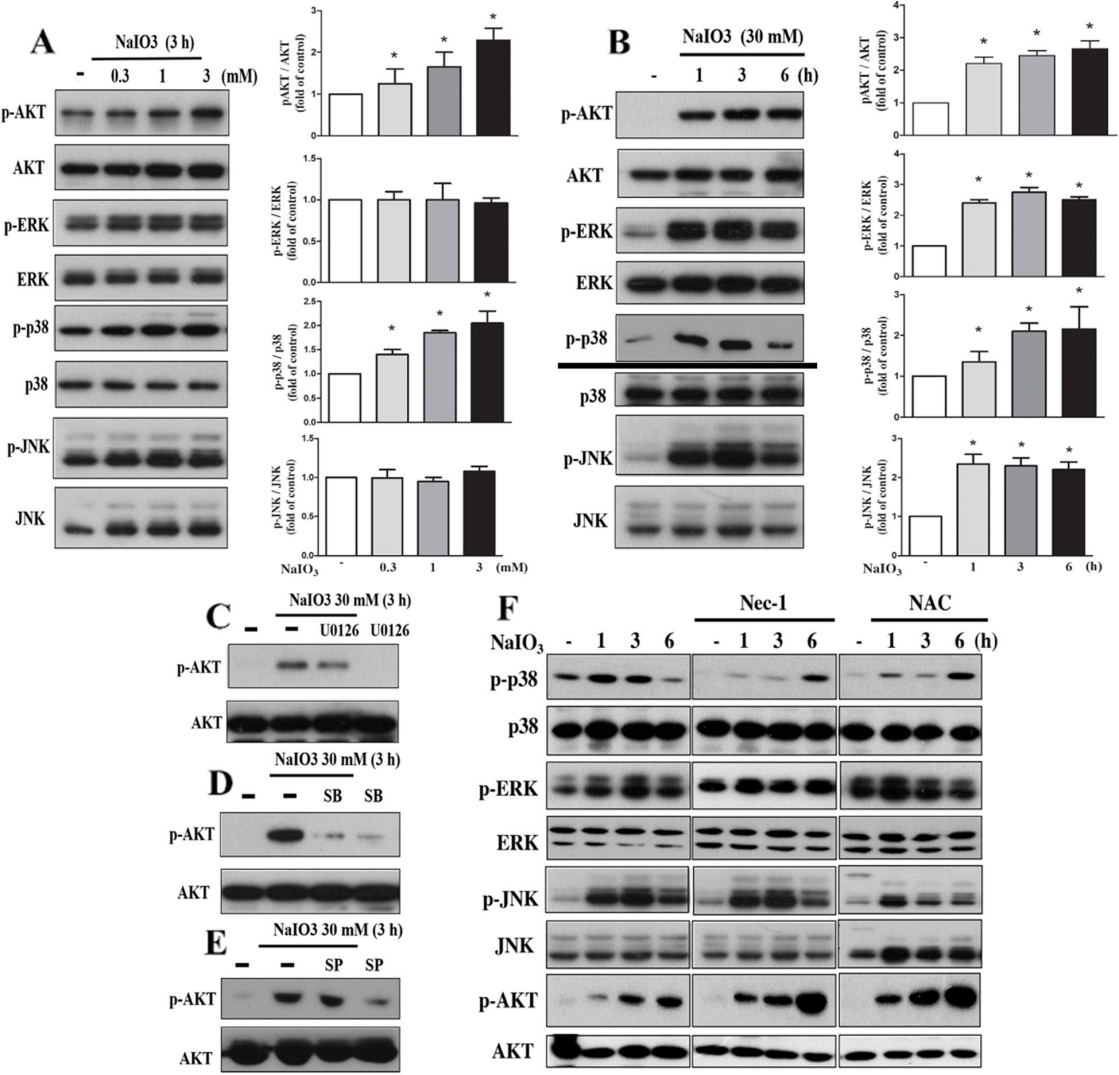
NaIO3 induces Akt, ERK, JNK and p38 MAPK activation. **a**, **b** As indicated, cells were treated with NaIO3 at concentrations indicated for different intervals. Immunoblotting was conducted by specific antibodies to determine the total and phosphorylated forms of Akt, ERK, p38 MAPK and JNK. Quantification of protein phosphorylation was determined by normalization with respective total protein levels. * *p* < 0.05, indicating significant activation effects of NaIO3 on signaling pathways. **c**-**f** Cells were pretreated with U0126 (ERK inhibitor, 10 μM) (**c**), SB203580 (p38 MAPK inhibitor, 10 μM) (**d**), SP600125 (JNK inhibitor, 10 μM) (**e**), Nec-1 (RIP1 inhibitor, 30 μM) (**f**) or NAC (10 mM) (**f**) for 15 min. Then cells were treated with NaIO3 (30 mM) for 3 h (**c**-**e**) or different time intervals (**f**). Cell lysates were collected for immunoblotting
